# Supplementary material for: How individual differences shape ERP responses to visual statistical learning
Source: bioRxiv. 2025 Aug 1:2025.07.30.667804. Preprint. [Version 1] doi: 10.1101/2025.07.30.667804 (PMC12324442; doi:10.1101/2025.07.30.667804)
Supplement: 1 [file NIHPP2025.07.30.667804v1-supplement-1.pdf]

bioRxiv preprint doi: https://doi.org/10.1101/2025.07.30.667804; this version posted August 1, 2025. The copyright holder for this preprint (which was not certified by peer review) is the author/funder, who has granted bioRxiv a license to display the preprint in perpetuity. It is made available under aCC-BY 4.0 International license.

Supporting information

S1 Table. Full model outputs for N100 and N400 ERP analyses. This table presents summary statistics for four linear mixed-effects models for each component (N100 and N400). It includes fixed effects, F-statistics, p-values, partial eta-squared effect sizes, marginal R^2, estimated marginal means (EMMs), standard errors, and Cohen's d with 95% confidence intervals for the familiarity effect across sensitivity conditions.

bioRxiv preprint doi: https://doi.org/10.1101/2025.07.30.667804; this version posted August 1, 2025. The copyright holder for this preprint (which was not certified by peer review) is the author/funder, who has granted bioRxiv a license to display the preprint in perpetuity. It is made available under aCC-BY 4.0 International license.

Table 1. Summary of linear mixed-effects model results

	N100						Familiar		Unfamiliar		Familiar - Unfamiliar			
Model	Effect	df	F	p	η^2 (partial)	R^2 (marginal)	EMM	SE	EMM	SE	Δ	SE	Cohen's d	95% CI
Model 1 -	Familiarity	1, 65	27.44	<.001	0.30	0.08	-3.55	0.51	-0.90	0.26	-2.65	0.51	-0.55	[-0.63, -0.47]
Dichotomised Sensitivity x Familiarity	Sensitivity	1, 65	2.5	0.119	0.04									
	Familiarity × Sensitivity	1, 65	1.03	0.313	0.02									
Model 2 -	Familiarity (sensitive)	1,16	9.44	0.007	0.37		-4.30	1.01	-1.13	0.53	-3.17	1.03	-0.63	[-0.79, -0.47]
Familiarity	Familiarity (Insensitive)	1,49	20.11	<.001	0.29		-2.80	0.48	-0.66	0.25	-2.14	0.48	-0.52	[-0.62, -0.43]
Model 3 - Continuous Sensitivity x Familiarity	Familiarity	1, 65	29.49	<.001	0.31	0.08	-3.18	0.44	-0.78	0.23	-2.40	0.44	-0.55	[-0.63, -0.47]
	Sensitivity	1, 65	2.04	0.158	0.03									
	Familiarity × Sensitivity	1, 65	1.36	0.249	0.02									
Model 4 -	Familiarity	1, 65	27.44	<.001	0.30	0.09	-3.55	0.51	-0.90	0.26	-2.65	0.51	-0.55	[-0.63, -0.47]
Dichotomised Sensitivity x Familiarity x Accuracy	Sensitivity	1, 65	2.50	0.119	0.04									
	Accuracy	1, 65	0.22	0.644	<.01									
	Familiarity × Sensitivity	1, 65	1.03	0.313	0.02									
	Sensitivity × Accuracy	1, 65	0.75	0.391	0.01									
	Familiarity × Accuracy	1, 65	8.44	0.004	<.01									
	Familiarity × Sensitivity × Accuracy	1,1673	56.01	<.001	0.03									
	N400						Familiar		Unfamiliar		Familiar - Unfamiliar			
Model	Effect	df	F	p	η^2 (partial)	R^2 (marginal)	EMM	SE	EMM	SE	Δ	SE	Cohen's d	95% CI
Model 1 -	Familiarity	1, 65	30.88	<.001	0.32	0.089	-1.92	0.63	2.06	0.58	-3.98	0.72	-0.61	[-0.7, -0.53]
Dichotomised Sensitivity x Familiarity	Sensitivity	1, 65	0.57	0.453	<.01									
	Familiarity × Sensitivity	1, 65	0.35	0.554	<.01									
Model 2 -	Familiarity (Sensitive)	1,16	22.06	<.001	0.58		-1.77	1.34	2.64	1.24	-4.4	0.94	-0.6	[-0.77, -0.44]
Familiarity	Familiarity (Insensitive)	1,49	21.3	<.001	0.30		-2.08	0.59	1.47	0.53	-3.55	0.77	-0.63	[-0.72, -0.53]
Model 3-	Familiarity	1, 65	36.37	<.001	0.36	0.087	-2.00	0.55	1.77	0.50	-3.77	0.62	-0.61	[-0.70, -0.53]
Continuous Sensitivity x Familiarity	Sensitivity	1, 65	0.26	0.614	<.01									
	Familiarity × Sensitivity	1, 65	0.22	0.64	<.01									
Model 4 -	Familiarity	1, 65	30.88	<.001	0.32	0.097	-1.92	0.63	2.06	0.58	-3.98	0.72	-0.55	[-0.63, -0.47]
Dichotomous Sensitivity x Familiarity x Accuracy	Sensitivity	1, 65	0.57	0.453	<.01									
	Accuracy	1, 65	1.13	0.291	0.02									
	Familiarity × Sensitivity	1, 65	0.35	0.554	<.01									
	Sensitivity × Accuracy	1, 65	0.32	0.572	<.01									
	Familiarity × Accuracy	1, 65	5.28	0.022	<.01									
	Familiarity × Sensitivity × Accuracy	1,1673	60.36	<.001	0.03									
